# Frequency of breast cancer subtypes among African American women in the AMBER consortium

**DOI:** 10.1186/s13058-018-0939-5

**Published:** 2018-02-06

**Authors:** Emma H. Allott, Joseph Geradts, Stephanie M. Cohen, Thaer Khoury, Gary R. Zirpoli, Wiam Bshara, Warren Davis, Angela Omilian, Priya Nair, Rochelle P. Ondracek, Ting-Yuan David Cheng, C. Ryan Miller, Helena Hwang, Leigh B. Thorne, Siobhan O’Connor, Traci N. Bethea, Mary E. Bell, Zhiyuan Hu, Yan Li, Erin L. Kirk, Xuezheng Sun, Edward A. Ruiz-Narvaez, Charles M. Perou, Julie R. Palmer, Andrew F. Olshan, Christine B. Ambrosone, Melissa A. Troester

**Affiliations:** 1 Department of Nutrition, University of North Carolina at Chapel Hill, Chapel Hill, NC USA; 2 Department of Pathology, Brigham and Women’s Hospital, Boston, MA USA; 3 Department of Pathology, Dana Farber Cancer Institute, Boston, MA USA; 4 Harvard Medical School, Boston, MA USA; 5 Lineberger Comprehensive Cancer Center, University of North Carolina at Chapel Hill, Chapel Hill, NC USA; 6 Translational Pathology Laboratory, University of North Carolina at Chapel Hill, Chapel Hill, NC USA; 7 Department of Pathology, Roswell Park Cancer Institute, Buffalo, NY USA; 8 Department of Cancer Prevention and Control, Roswell Park Cancer Institute, Buffalo, NY USA; 9 Massachusetts General Hospital, Boston, MA USA; 10 Department of Epidemiology, University of Florida, Gainesville, FL USA; 11 Department of Pathology and Laboratory Medicine, University of North Carolina at Chapel Hill, Chapel Hill, NC USA; 12 Department of Pathology, University of Texas Southwestern, Dallas, TX USA; 13 Slone Epidemiology Center at Boston University, Boston, MA USA; 14 Department of Medicine, Boston University School of Medicine, Boston, MA USA; 15 Department of Epidemiology, CB 7435, University of North Carolina at Chapel Hill, 135 Dauer Drive, Chapel Hill, NC 27599 USA

**Keywords:** African American, Automated digital pathology, Basal-like, Immunohistochemistry, Luminal, PAM50

## Abstract

**Background:**

Breast cancer subtype can be classified using standard clinical markers (estrogen receptor (ER), progesterone receptor (PR) and human epidermal growth factor receptor 2 (HER2)), supplemented with additional markers. However, automated biomarker scoring and classification schemes have not been standardized. The aim of this study was to optimize tumor classification using automated methods in order to describe subtype frequency in the African American Breast Cancer Epidemiology and Risk (AMBER) consortium.

**Methods:**

Using immunohistochemistry (IHC), we quantified the expression of ER, PR, HER2, the proliferation marker Ki67, and two basal-like biomarkers, epidermal growth factor receptor (EGFR) and cytokeratin (CK)5/6, in 1381 invasive breast tumors from African American women. RNA-based (prediction analysis of microarray 50 (PAM50)) subtype, available for 574 (42%) cases, was used to optimize classification. Subtype frequency was calculated, and associations between subtype and tumor characteristics were estimated using logistic regression.

**Results:**

Relative to ER, PR and HER2 from medical records, central IHC staining and the addition of Ki67 or combined tumor grade improved accuracy for classifying PAM50-based luminal subtypes. Few triple negative cases (< 2%) lacked EGFR and CK5/6 expression, thereby providing little improvement in accuracy for identifying basal-like tumors. Relative to luminal A subtype, all other subtypes had higher combined grade and were larger, and ER-/HER2+ tumors were more often lymph node positive and late stage tumors. The frequency of basal-like tumors was 31%, exceeded only slightly by luminal A tumors (37%).

**Conclusions:**

Our findings indicate that automated IHC-based classification produces tumor subtype frequencies approximating those from PAM50-based classification and highlight high frequency of basal-like and low frequency of luminal A breast cancer in a large study of African American women.

**Electronic supplementary material:**

The online version of this article (10.1186/s13058-018-0939-5) contains supplementary material, which is available to authorized users.

## Background

Breast cancer comprises several tumor subtypes with distinct etiologies and clinical outcomes [1, 2]. However, high assay cost and limited amounts of archived tumor tissue may prevent utilization of RNA-based (e.g. prediction analysis of microarray 50 (PAM50)) subtype classification methods in epidemiologic studies. Surrogate immunohistochemistry (IHC)-based subtype classification schemes are widely used, and even emphasized in St. Gallen guidelines [3]. Specifically, quantitative IHC for Ki67 and progesterone receptor (PR) is recommended for classification of luminal (estrogen receptor (ER) positive) subtypes, while cytokeratin (CK) 5/6 and epidermal growth factor receptor (EGFR) are recommended to accurately identify basal-like breast cancers among tumors that are negative for all three standard clinical markers (ER, PR, and human epidermal growth factor receptor 2 (HER2)). However, thresholds for categorizing these IHC-based biomarkers have been predominantly selected based on clinical samples, and have not been optimized for epidemiologic studies or for studies using automated digital pathology approaches for tumor subtyping.

We quantified the expression of six tumor biomarkers using automated methods for scoring IHC staining of tissue microarrays (TMAs) comprising 1381 cases of invasive breast cancer in African American (AA) women from the African American Breast Cancer Epidemiology and Risk (AMBER) consortium [4]. The aim of this study was to optimize IHC-based tumor classification with respect to PAM50-based subtype, and to describe the frequency and characteristics of breast cancer subtypes in the AMBER consortium.

## Methods

### Study population

This analysis is based on data from 1552 breast cancer cases in the AMBER consortium [4] for which paraffin-embedded tumor tissue was available on TMAs. Cases were from the Carolina Breast Cancer Study phase 3 (CBCS, n = 819), the Black Women’s Health Study (BWHS, n = 326) and the Women’s Circle of Health Study (WCHS, n = 407). The CBCS was approved by the Institutional Review Board at the University of North Carolina at Chapel Hill School of Medicine. The BWHS was approved by the Institutional Review Board at the Boston University School of Medicine. The WCHS was approved by the Institutional Review Boards at the University of Medicine and Dentistry of New Jersey (presently Rutgers University), Mount Sinai School of Medicine, and Roswell Park Cancer Institute. Written informed consent was obtained from each participant. Combined grade, tumor size, lymph node status, and tumor stage were abstracted from medical records, and these tumor characteristics were available for 98%, 100%, 97%, and 98% of all study participants, respectively. Combined grade was also centrally assigned by a breast pathologist (JG for CBCS; HH and TK for WCHS and BWHS) using the Nottingham breast cancer grading system [5], and was available for 96% of cases.

### Immunohistochemistry staining and quantification

Paraffin-embedded tumor blocks were requested from clinical pathology facilities, and TMA construction and sectioning was carried out for CBCS at the Translational Pathology Lab (TPL), University of North Carolina at Chapel Hill (UNC) and at Roswell Park Cancer Institute (RPCI) for BWHS and WCHS [6]. All central IHC staining was performed at the UNC TPL; detailed methods for ER, PR and HER2 have been described [6], and are provided in Additional file 1: Supplementary Methods for Ki67, EGFR and CK5/6. Automated quantification of IHC staining was performed using a Genie classifier and Nuclear v9 (for ER, PR, and Ki67) and Membrane v9 (for HER2, EGFR, and CK5/6) algorithms (Aperio Technologies, Vista, CA, USA) [6]. For all six biomarkers, the Genie classifier was used to eliminate regions of folded tissue and other artifacts to reduce false positives. For ER, PR and HER2, the Genie classifier was used to exclude stromal cells, thereby enriching for tumor epithelium. For CK5/6, the Genie classifier was designed to reduce the number of positive myoepithelial cells included in the analysis.

### Immunohistochemistry-based biomarker thresholds

We used previously described core-to-case collapsing methods to define biomarker status [6]. For ER, PR, HER2, and Ki67, average biomarker expression across all cores for a given case was weighted by the cellularity of each core. For EGFR and CK5/6, we assigned positive status to the case if any core was positive, given that these biomarkers are more heterogeneously expressed than ER and PR [7]. Indeed, manual review of 26 PAM50-defined basal-like tumors revealed heterogeneous expression of CK5/6 or EGFR in 10 (38%) cases, whereas our prior work identified manually confirmed ER, PR, or HER2 heterogeneity in < 10% of cases [7].

A 10% threshold for ER and PR biomarker expression was applied to maximize agreement with RNA-based intrinsic subtype and with medical records, as previously published in the AMBER consortium [6]. We explored a 20% threshold to classify PR status, as recommended by St. Gallen guidelines [3] based on work by Prat et al. [8]. We identified an optimal Ki67 threshold by generating a receiver operating characteristic (ROC) curve among HER2-negative luminal tumors and applying the Youden method [9] to maximize the sum of the sensitivity and specificity for PAM50-defined luminal B tumors (Additional file 2: Figure S1). This method identified a threshold of 7.6%, and we rounded this threshold to the nearest integer (8%). We repeated ROC curve analysis among all luminal cases regardless of IHC-based HER2 status, identifying an optimal Ki67 threshold of 7.1%. We applied ≥ 1% thresholds to classify EGFR and CK5/6 status, given that previous studies recommended that EGFR and CK5/6 expression be defined as any positive staining [10, 11]. We validated this threshold using manual review of a subset of 26 PAM50-defined basal-like cases, finding that automated scoring correctly classified basal-like biomarker expression in 25 of 26 (> 96%) manually reviewed PAM50-defined basal-like cases (data not shown). In exploratory analysis, we generated ROC curves among IHC-based triple negative cases to select study-specific EGFR and CK5/6 thresholds for identifying PAM50-defined basal-like tumors. We found a 2% CK5/6 threshold to be optimal for identifying basal-like breast cancer in the AMBER consortium, while EGFR expression did not distinguish triple negative basal-like cases from triple negative cases that were not basal-like (data not shown).

Of 1552 cases in total, 83 (5%) were missing one or more biomarkers such that IHC-based subtype could not be defined. A further 81 (5%) had equivocal (2+) HER2 status and were therefore unable to be classified, leaving a total of 1381 cases with IHC-based subtype for analysis.

### RNA-based subtyping

Nanostring assays were used to measure the PAM50 gene signature in 488 cases from CBCS and 145 cases from BWHS, and were performed in the Rapid Adoption Molecular laboratory at UNC. For CBCS, two 1.0-mm tumor cores from the tumor block used for TMA construction were sampled within tumor regions circled by a study pathologist (J. Geradts or L.B. Thorne) and pooled for analysis. The areas surrounding the holes left by the cores were subsequently examined by a study pathologist to confirm high tumor cellularity in the cores used for RNA extraction. For BWHS, 10-μm paraffin sections on uncharged slides were scraped for analysis. The PAM50 predictor was performed as previously described [12] to classify tumors into intrinsic subtypes (luminal A, luminal B, HER2-enriched, basal-like, normal-like). Of 1381 cases with IHC-based subtype, 574 (40%) also had RNA-based PAM50 subtype (n = 449 CBCS cases and n = 125 BWHS cases). Tumors classified as normal-like (n = 22) were treated as missing PAM50 subtype, given that this classification is thought to arise from extensive normal epithelial or stromal content in the tumor [13]. Indeed, we found that median tumor cellularity was significantly lower among normal-like cases than other subtypes (2464 vs. 5543 cells per core; rank-sum test *p* < 0.001). Relative to cases without PAM50 data, cases with PAM50 data were younger at diagnosis, had larger tumors, higher combined grade and higher tumor stage and were more likely to be ER-negative and PR-negative; there were no differences in lymph node or HER2 status.

### Statistical analysis

Kernel density plots of Ki67 and PR expression in PAM50-defined luminal A and luminal B subtypes were constructed, overall and restricted to tumors that were HER2-negative by IHC. We compared sensitivity (true positive/(true positive + false negative)), specificity (true negative/(true negative + false positive)) and accuracy ((true positive + true negative)/total cases) of IHC-based classification schemes for identifying luminal A and luminal B PAM50-based subtypes.

We examined the frequency of IHC-based subtypes in the AMBER consortium overall and across contributing studies. Our findings were similar whether or not CBCS subtype frequencies were weighted for sampling scheme, and we chose to present weighted percentages. Multinomial logistic regression was used to generate odds ratios (ORs) and 95% confidence intervals (CIs) for associations between age, menopause status, and IHC-based subtype, treating luminal A cases as the referent group. We also used multinomial logistic regression to examine differences in tumor characteristics across IHC-based subtypes. In sensitivity analysis, we adjusted these models for study site. Statistical analyses were conducted using STATA version 13.1 (Stata Corp., College Station, TX, USA).

## Results

### Immunohistochemistry-based classification of non-basal-like breast cancer

Subtype classification using three biomarkers (ER, PR, and HER2) produced high sensitivity for luminal A (82%), but low sensitivity for luminal B tumors (20%; Table 1). The addition of combined tumor grade substantially increased sensitivity for classification of luminal B tumors, resulting in improved overall accuracy for both luminal A (81% with grade vs. 73% without) and luminal B classification (81% with grade vs. 79% without). Similar gains in accuracy were observed when adding combined grade to three biomarker medical record-based classification, although the accuracy of central IHC-based classification was slightly better than medical record-based classification overall (Additional file 3: Table S1).

**Table 1:** Classification of luminal breast cancer cases using data from central immunohistochemistry assays in the AMBER consortium

		Subtype frequency, *n* (%)	Sensitivity for PAM50 subtype	Specificity for PAM50 subtype	Accuracy for PAM50 subtype
HR/HER2					
Luminal A	HR+/HER2-	606 (50)	82%	69%	73%
Luminal B	HR+/HER2+	140 (11)	20%	94%	79%
HR/HER2/combined grade					
Luminal A	HR+/HER2-, low/intermediate grade	448 (37)	69%	86%	81%
Luminal B	HR+/HER2+, *or* HR+/HER2-, high grade	298 (24)	64%	85%	81%
HR/HER2/Ki67					
Luminal A	HR+/HER2-, low Ki67^a^	420 (34)	66%	87%	80%
Luminal B	HR+/HER2+, *or* HR+/HER2-, high Ki67^a^	326 (27)	68%	84%	81%
HR/Ki67					
Luminal A	HR+, low Ki67^b^	453 (37)	67%	86%	80%
Luminal B	HR+, high Ki67^b^	293 (24)	68%	85%	82%

*HR* hormone receptor, *PAM50* prediction analysis of microarray 50, *HER2* human epidermal growth factor receptor 2

^a^Ki67 threshold of 8%, as identified by ROC analysis among HER2-negative luminal tumors

^b^Ki67 threshold of 7%, as identified by ROC analysis among all luminal tumors

St. Gallen guidelines recommend the use of quantitative PR and Ki67 for classification of hormone receptor-positive, HER2-negative tumors [3]. However, we found similar PR expression levels in HER2-negative luminal A and B tumors (Additional file 4: Figure S2) and, relative to a 10% threshold, a 20% PR threshold (as recommended by St. Gallen guidelines [3]) had reduced accuracy for identifying luminal tumors (data not shown). In contrast, the addition of Ki67 improved accuracy for identifying luminal tumors relative to three biomarkers alone (80% vs. 73% for luminal A, and 81% vs. 79% for luminal B tumors; Table 1), comparable to the effect of adding combined grade.

Recent data show that not all luminal A tumors are HER2-negative [14]. Indeed, 9–11% of PAM50-based luminal A tumors in the AMBER consortium were HER2-positive (based on HER2 status from central IHC staining and medical records, respectively). As such, we removed HER2 from the luminal classification scheme and this produced very similar, if slightly improved, accuracy for identifying PAM50-defined luminal subtypes, relative to the three biomarker + Ki67 scheme (Table 1). Finally, we explored the higher Ki67 thresholds used in other studies [3, 15], but this reduced sensitivity for luminal B cases and decreased overall accuracy for distinguishing luminal subtypes in the AMBER consortium, illustrating the importance of study-specific Ki67 thresholds as recommended by St. Gallen guidelines (Additional file 5: Table S2).

**Fig. 1:**
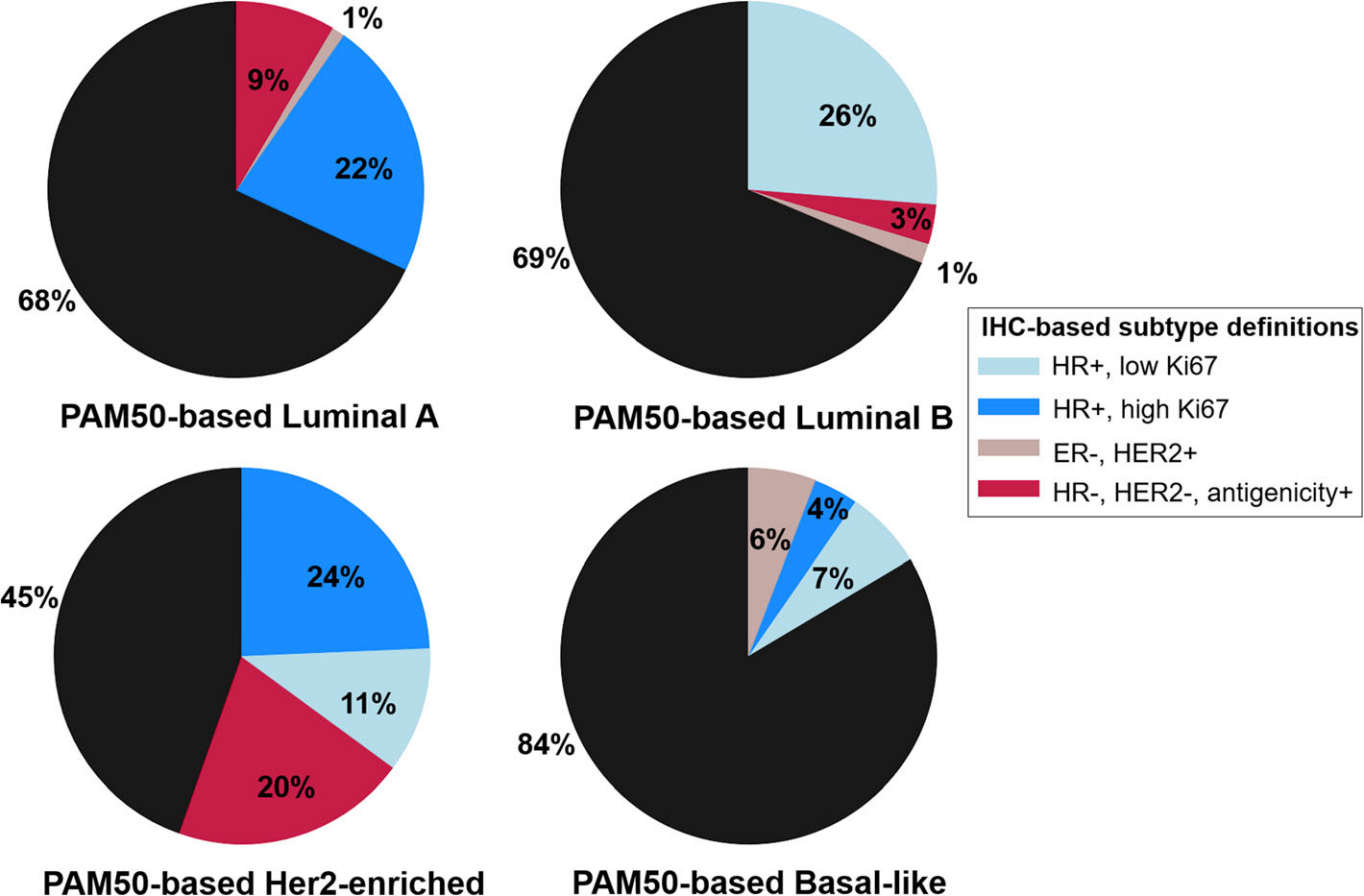
Frequency of immunohistochemistry (IHC)-based subtypes within prediction analysis of microarray 50 (PAM50)-based subtype categories. Black pie slices represent the percentage of each PAM50-based subtype correctly identified using IHC-based definitions, while colored slices represent IHC-based subtypes of misclassified cases. HR, hormone receptor; ER, estrogen receptor; HER2, human epidermal growth factor receptor 2

Additional biomarkers for accurate IHC-based classification of HER2-enriched tumors have not been identified. Therefore, we applied the standard definition of ER-/HER2+, which yielded 45% sensitivity, 97% specificity, and 90% accuracy (Fig. 1), in line with our previous findings [6]. Given the low sensitivity for PAM50-based HER2-enriched tumors using this classification scheme, we refer to this subtype as ER-/HER2+.

### Immunohistochemistry-based classification of basal-like breast cancer

IHC-based classification using triple negative status alone (ER-, PR-, and HER2-) produced high sensitivity for PAM50-defined basal-like breast cancer (84%; Fig. 1 and Table 2). The addition of EGFR and CK5/6 did not affect accuracy, as extremely few triple negative cases (n = 7; < 2%) lacked expression of both basal-like biomarkers. Relative to triple negative status with ≥ 1% EGFR or CK5/6 expression, the use of a 2% CK5/6 threshold without EGFR resulted in higher specificity (91% vs. 96%, respectively) but lower sensitivity for basal-like tumors (83% vs. 53%, respectively), and produced a relatively large proportion of triple negative tumors that were not basal-like (41% of all triple negative cases; Table 2). Given that the majority (83%) of triple negative tumors in the AMBER consortium was classified as basal-like using PAM50, we proceeded with the 1% threshold for EGFR or CK5/6 expression in order to maximize sensitivity and overall accuracy for PAM50-based basal-like breast cancer, and to limit the number of tumors misclassified as triple negative non-basal-like.

**Table 2:** Classification of basal-like breast cancer cases using data from central immunohistochemistry assays in the AMBER consortium

IHC-based basal-like classification scheme	Number (percentage) of PAM50 basal-like tumors identified by IHC	Number (percentage) of unclassified (i.e. triple negative, non-PAM50 basal-like)^c^	Sensitivity for PAM50 subtype	Specificity for PAM50 subtype	Accuracy for PAM50 subtype
HR-, HER2-	174 (84)	-	84%	90%	88%
HR-, HER2-, (EGFR+ or CK5/6+)^a^	173 (83)	3 (1.4)	83%	91%	88%
HR-, HER2-, CK5/6+^b^	110 (53)	86 (41)	53%	96%	81%

*IHC* immunohistochemistry, *HR* hormone receptor, *HER2* human epidermal growth factor receptor 2, *EGFR* epidermal growth factor receptor, *CK5/6* cytokeratin 5/6, *PAM50* prediction analysis of microarray 50

^a^EGFR+ and CK5/6+ defined as ≥ 1% staining in any core

^b^CK5/6+ defined as a weighted average ≥2% across all cores for a given case

^c^Of 210 IHC-based triple negative cases with PAM50 subtype

### Frequency and characteristics of six-biomarker immunohistochemistry-based breast cancer subtypes

Using the optimized six biomarker IHC-based subtype classification scheme, the frequency of basal-like breast cancer in the AMBER consortium was 31%, while the frequency of luminal A and luminal B cancers was 37% and 25%, respectively (Table 3). ER-/HER2+ cancers comprised 8% of all cases. Luminal A cancers were more frequent in the BWHS than in the CBCS (46% vs. 32% luminal A) while luminal B and basal-like cancers were more frequent in the CBCS than in the BWHS (29% vs. 17% luminal B; 33% vs. 28% basal-like).

**Table 3:** Frequency of six-marker immunohistochemistry-defined subtypes^a^, overall and by study site in the AMBER consortium

	IHC-based definition^a^	Overall *n* (%)	CBCS *n* (%)	WCHS *n* (%)	BWHS *n* (%)
Luminal A	HR ≥10%, Ki67 <7%	512 (37)	233 (32)	146 (42)	133 (46)
Luminal B	HR ≥10%, Ki67 ≥7%	333 (25)	212 (29)	73 (21)	48 (17)
ER-/HER2+	ER <10%, HER2+	111 (8)	52 (7)	32 (9)	27 (9)
Basal-like	HR <10%, HER2-, antigenicity+	425 (31)	247 (33)	99 (28)	79 (28)
Total		1381	744	350	287
Unclassified^b^		171	75	57	39

CBCS percentages are weighted for study sampling scheme

*ER* estrogen receptor, *HER2* human epidermal growth factor receptor 2, *IHC* immunohistochemistry, *HR* hormone receptor, *CBCS* Carolina Breast Cancer Study, *WCHS* Women’s Circle of Health Study, *BWHS* Black Women’s Health Study

^a^Antigenicity+ = CK5/6 ≥1% or EGFR ≥1%

^b^Tumors with five-marker negative (n = 7), HER2-equivocal (n = 81), and missing biomarker (n = 83) status remained unclassified

The frequency of luminal B tumors in the AMBER consortium did not differ from that of luminal A tumors with respect to age or menopausal status at diagnosis (Table 4). However, ER-/HER2+ and basal-like cancers were significantly more frequent at younger ages. Results were similar when ORs were adjusted for AMBER study site (Additional file 6: Table S3).

**Table 4:** Differences in age and menopause^a^ status at diagnosis across six-marker immunohistochemistry-defined subtypes^b^ in the AMBER consortium

	Age ≥50 years, *n* (%)	Age <50 years,*n* (%)	OR (95% CI)	Postmeno, *n* (%)	Premeno, *n* (%)	OR (95% CI)
Luminal A	305 (39)	207 (34)	1	300 (39)	204 (35)	1
Luminal B	195 (25)	138 (23)	1.04 (0.79–1.38)	194 (25)	135 (23)	1.02 (0.77–1.36)
ER-/HER2+	49 (6)	62 (10)	1.86 (1.23–2.82)	51 (7)	57 (10)	1.64 (1.08–2.30)
Basal-like	224 (29)	201 (33)	1.32 (1.02–1.71)	233 (30)	182 (31)	1.15 (0.88–1.49)
*P value* ^*c*^	*0.008*			*0.107*		
Unclassified	93	78		93	72	

Tumors with five-marker negative (n = 7), human epidermal growth factor 2 (HER2)-equivocal (n = 81), and missing biomarker (n = 83) status remained unclassified

*ER* estrogen receptor, *Postmeno* postmenopausal, *Premeno* premenopausal

^a^There were 25 cases with missing data on menopause status

^b^Luminal A: HR+, low Ki67; luminal B: HR+, high Ki67; ER-/HER2+: ER <10% and HER2-positive; basal-like: HR-, HER2- and (EGFR+ *or* CK5/6+)

^c^Chi-square test *p* value excludes unclassified cases

Relative to luminal A breast cancer, all other subtypes had higher combined grade and were larger (Table 5). However, only ER-/HER2+ tumors were later stage and more likely to be lymph node positive, relative to luminal A tumors.

**Table 5:** Tumor characteristics associated with six-marker immunohistochemistry-based subtypes^a^ in the AMBER consortium

IHC-based subtype	Number	High combined grade(3 vs. 1/2)		Large tumor size (>2 vs. ≤2 cm)		Positive lymph node status (positive vs. negative)		High stage (III/IV vs. I/II)	
		*n* (%)	OR (95% CI)	*n* (%)	OR (95% CI)	*n* (%)	OR (95% CI)	*n* (%)	OR (95% CI)
Luminal A	512	78 (16)	*1.00 (ref)*	198 (39)	*1.00 (ref)*	196 (39)	*1.00 (ref)*	69 (14)	*1.00 (ref)*
Luminal B	333	158 (48)	4.99 (3.61–6.91)	152 (46)	1.33 (1.01–1.76)	130 (40)	1.03 (0.77–1.37)	44 (14)	0.98 (0.66–1.48)
ER-/HER2+	111	86 (82)	24.02 (13.82–41.75)	57 (51)	1.67 (1.11–2.53)	62 (58)	2.16 (1.41–3.31)	26 (24)	2.02 (1.21–3.36)
Basal-like	425	365 (90)	46.13 (30.90–68.86)	252 (59)	2.31 (1.78–3.00)	173 (41)	1.09 (0.84–1.42)	62 (15)	1.10 (0.76–1.60)

*IHC* immunohistochemistry, *ER* estrogen receptor, *HER2* human epidermal growth factor receptor 2

^a^Luminal A: hormone receptor (HR)+, HER2-, low Ki67; luminal B: (HR+, HER2+) *or* (HR+, HER2-, high Ki67); ER-/HER2+: ER <10% and HER2-positive; basal-like: HR-, HER2- and (EGFR+ *or* CK5/6+)

Tumors with five-marker negative (n = 7), HER2-equivocal (n = 93), and missing biomarker (n = 99) status excluded from analysis

## Discussion

Accurate classification of tumor subtype is critical for understanding clinical and etiologic heterogeneity in breast cancer. Using automated methods to score central biomarker data from 1381 cases among African Americans (AAs) in the AMBER consortium, we optimized IHC-based tumor classification to maximize sensitivity, specificity, and accuracy with respect to PAM50 subtype. Implementing our optimized IHC-based classification scheme, we report a high frequency of basal-like breast cancer in the AMBER consortium (31% of all cases), suggesting that the frequency of this subtype in AAs is similar to that of luminal A and luminal B tumors (37% and 25%, respectively). The frequency of luminal A tumors was overestimated (55%) when relying on ER, PR and HER2 from medical records alone, underscoring the importance of using central IHC staining and additional markers, such as Ki67 or grade, to accurately classify luminal tumors. Overall, this work highlights the use of automated IHC-based methods to approximate PAM50-based subtype frequencies and confirms a high prevalence of basal-like breast cancer among AA women in a large consortium.

Accurately distinguishing luminal A from luminal B breast cancer, subtypes with distinct clinical outcome and potentially different etiology, is a significant challenge in epidemiologic studies. Moreover, because luminal A cases often serve as the reference group in case-only analyses [1, 16], improving accuracy for identifying luminal A tumors is critical for etiologic and survivorship studies of all subtypes. Prat and colleagues reported that luminal A tumors could be identified by their substantial (> 20%) expression of PR [8], leading to the incorporation of quantitative PR data into St. Gallen guidelines [3]. However, this observation was not replicated in our study, suggesting that PR may not reliably segregate luminal A and B tumors across different populations. On the other hand, we found that incorporating Ki67 data, as recommended by St. Gallen guidelines, improved accuracy for luminal tumor classification in the AMBER consortium. Moreover, the use of an automated scoring algorithm afforded more precision in selecting Ki67 thresholds, compared to manually estimating biomarker thresholds. However, one challenge with utilizing Ki67 data is the necessity of establishing study-specific standards [3]; the 7% Ki67 threshold optimized for the AMBER study is lower than in other studies that used 14% or 20% thresholds [15, 17]. Automated biomarker quantification methods, as used in AMBER, calculate biomarker expression among a range of cell types within a tumor, while manual review used by other studies may exclude benign epithelium, immune infiltrates, or stromal cells more accurately [7]. However, we enriched for tumor epithelium in the AMBER consortium by excluding tissue microarray (TMA) cores with low tumor cellularity, and so it may be that Ki67 staining protocols and scoring algorithms are merely difficult to standardize across studies. Indeed, an international working group evaluating inter-laboratory reproducibility for Ki67 showed that this biomarker is challenging to harmonize, even across some of the world’s most experienced laboratories [18]. Confidence in our Ki67 threshold can be derived from its optimization with respect to RNA-based subtype, while prior studies selected study-specific Ki67 thresholds based on clinical outcome [19, 20]. Given that both approaches have merit, researchers should be guided by whether the goal is to study breast cancer etiology or breast cancer outcomes. Importantly, our data suggest that combined tumor grade, taken either from the clinical record or determined centrally, distinguishes luminal tumors with similar accuracy to Ki67. Thus, tumor grade could be used in epidemiologic studies that do not have access to Ki67 data. Finally, findings from the AMBER and other studies [14, 15] show that approximately 70% of PAM50-defined luminal B tumors lack HER2 protein expression, while approximately 10% of PAM50-defined luminal A tumors express HER2, suggesting that HER2 may not be useful for distinguishing luminal subtypes. Indeed, we found that dropping HER2 from our IHC-based classification scheme produced similar, if slightly improved, accuracy for identifying luminal A and luminal B tumors.

Subtype classification based on absence of biomarker expression is often deemed unreliable, and best practice has dictated identifying a positive marker for each tumor subtype. As such, expression of either EGFR or CK5/6 has been proposed for classification of basal-like breast cancer [3]. Of 474 cases of triple negative breast cancer in the AMBER consortium, only 7 (< 2%) lacked expression of both basal-like markers. This finding is in marked contrast to previous studies, some of which reported that up to 40% of triple negative cases are negative for both EGFR and CK5/6 [10, 11, 21]. However, adjusting EGFR and CK5/6 thresholds in the AMBER consortium to produce similar rates of triple negative non-basal-like cases resulted in the misclassification of almost half of all PAM50-defined basal-like cases as triple negative non-basal-like, and this would likely impede our ability to conduct adequately powered analyses of basal-like etiology and survivorship patterns in the AMBER consortium. An important distinction between previous studies and our own is that previous studies manually assessed IHC-based EGFR and CK5/6 expression [10, 11] and therefore counted only EGFR-positive and CK5/6-positive tumor cells. We considered a case to be antigen-positive when either the tumor or the surrounding normal epithelium or stroma was positive for either one of these markers. However, manual review of a subset of PAM50-based basal-like cases revealed extremely high agreement (>96%) between manual and automated scoring of basal-like markers. An alternative explanation for the discrepancy in number of triple negative non-basal-like cases lies in the significant decline in antigenicity of cut sections after several months to one year of storage at room temperature, potentially contributing to false negative biomarker status in studies reporting higher percentages of quintuple negative tumors [22, 23]. We maximized tissue antigenicity in the AMBER study by using a nitrogen desiccation chamber for storage of unstained slides. Finally, based on high intratumoral heterogeneity for CK5/6 and EGFR, when using TMAs our results support interpretation of these biomarkers as antigenicity markers, such that any positivity should support classification of ER, PR, and HER2 negative samples as basal-like. In sum, given that a biomarker robustly expressed by all basal-like tumors has not yet been identified, interpretation of CK5/6 and EGFR as markers of tissue antigenicity may be reasonable and, in our hands, yielded the highest sensitivity for detecting PAM50-defined basal-like tumors.

Our study has both strengths and weaknesses. First, we used data from TMAs comprising different core diameters (0.6 mm and 1.0 mm). This approach may introduce technical sources of variability in biomarker expression and affect the selection of biomarker thresholds. However, we previously explored multiple sources of technical variability [7], and optimized our IHC quantification methods accordingly. We also strengthened our analysis through validation of automated staining protocols guided by pathologists, and through optimization of IHC-based classification using RNA-based multigene assays. Our prior study [6], together with our unpublished observations, provide reassurance that biomarker thresholds and subtype classification schemes described here are appropriate for both white and African American breast cancer cases in the CBCS, one of the three studies contributing to the AMBER consortium. As such, we believe that our approach, with proper validation and methodological work warranted in distinct study resources, is generalizable to other studies using automated methods to classify breast cancer subtype in racially diverse populations. It is noteworthy that associations between subtype and tumor characteristics in the AMBER consortium were similar to those reported previously, albeit slightly stronger [24], perhaps due to higher specificity/greater purity of the luminal A reference group with the addition of Ki67 expression data. Relative to luminal A tumors in the AMBER consortium, all other subtypes had higher combined grade and were larger, but only ER-/HER2+ tumors were more likely to be lymph node positive and later-stage tumors. Stage at diagnosis did not differ between luminal A and basal-like tumors, a finding in line with SEER data [24]. These associations with tumor characteristics underscore that basal-like tumors tend to have more aggressive characteristics, while luminal A tumors tend to be more indolent. These associations have been relatively consistent across studies and in different racially defined subpopulations [25–27].

Using central biomarker data in this consortium, we showed that the frequency of basal-like breast cancer ranged from 28% to 33% across contributing studies, consistent with past studies in AAs using IHC-based subtype classification [28–30] and PAM50 assays [31]. These frequency estimates for basal-like tumors are consistently higher than those reported in white subjects, which range from 8% to 12% [24, 26, 31]. Conversely, luminal A tumors are less frequent in AAs relative to white subjects, comprising 32–46% of breast cancer cases across the AMBER studies. A smaller study of approximately 150 AA women reported a similar frequency of luminal A tumors [31], while > 50% of all breast tumors in white women are luminal A tumors [26, 31]. Lower rates of screening have been documented in studies of AA women [32], potentially contributing to lower detection rates for the more indolent luminal A breast cancers in AAs relative to white women. Screening data were not collected in the AMBER studies, and so we were unable to consider the effect of mode of detection on subtype frequency in the present study. However, the work of our group and others has identified etiologic factors associated with ER-negative and basal-like breast cancer [16, 33–35], some of which are differentially distributed by race [36, 37]. Although beyond the scope of the present study, continued analyses of etiologic exposure and mode of detection in the context of accurate subtyping are needed to better understand the underlying causes of breast cancer racial disparities. Upon completion, the AMBER consortium will comprise > 4000 cases of breast cancer in AAs, with banked tumor tissue and > 4000 AA controls, with extensive risk factor data harmonized. With approaches to classifying tumor subtypes now well established in the AMBER consortium, we will be able to better understand the underlying etiology of more aggressive breast cancers in AA women.

## Conclusions

In summary, using PAM50-validated IHC-based tumor classification, we provide the largest dataset to date on the frequency of breast cancer subtypes in AA women. Our findings validate the use of automated IHC-based methods to approximate PAM50-based subtype frequencies and highlight high frequency of basal-like and low frequency of luminal A breast cancer in a large consortium of AA women.

## Additional files

Additional file 1: Supplementary methods. (DOCX 15 kb)

Additional file 2: Figure S1. Receiver operating characteristic (ROC) curve in 250 HER2-negative luminal cases (**A**) and 309 luminal cases (**B**) regardless of HER2 status, using Ki67 expression to identify PAM50-based luminal B cases. A Ki67 threshold of 7.6% among HER2-negative luminal cases (**A**) and 7.1% among all luminal cases (**B**) maximized sensitivity and specificity for identifying PAM50-based luminal B tumors. AUC, area under the curve. (DOCX 43 kb)

Additional file 3: Table S1. Classification of luminal breast cancer cases using data from medical records in the AMBER consortium. (DOCX 12 kb)

Additional file 4: Figure S2. Kernel density plots showing the distribution of Ki67 and PR expression (**A** and **B**, respectively) in PAM50-defined luminal A (n = 159) and luminal B (n = 91) subtypes. Analyses were restricted to IHC-based HER2-negative tumors. Vertical lines indicate an 8% threshold for Ki67 (**A**) and a 20% threshold for PR (**B**). (DOCX 56 kb)

Additional file 5: Table S2. Effect of raising the Ki67 threshold on the performance of the IHC-based luminal classification scheme. (DOCX 12 kb)

Additional file 6: Table S3. Odds ratios and 95% confidence intervals for age and menopause^b^ status at diagnosis by six-marker immunohistochemistry-defined subtype^a^ in the AMBER consortium. (DOCX 13 kb)

## Abbreviations

AA: African American
AMBER: African American Breast Cancer Consortium
BWHS: Black Women’s Health Study
CBCS: Carolina Breast Cancer Study
CK5/6: Cytokeratin 5/6
EGFR: Epidermal growth factor receptor
ER: Estrogen receptor
HER2: Human epidermal growth factor receptor 2
HR: Hormone receptor
IHC: Immunohistochemistry
PAM50: prediction analysis of microarray 50
PR: Progesterone receptor
TMA: Tissue microarray
TPL: Translational Pathology Lab
UNC: University of North Carolina at Chapel Hill
WCHS: Women’s Circle of Health Study
