# Supplementary material for: Frequency of breast cancer subtypes among African American women in the AMBER consortium
Source: Breast Cancer Res. 2018 Feb 6;20:12. doi: 10.1186/s13058-018-0939-5 (PMC5801839; doi:10.1186/s13058-018-0939-5)
Supplement: Supplementary file 1 — Supplementary methods. (DOCX 15 kb) [file 13058_2018_939_MOESM1_ESM.docx]

*Immunohistochemistry staining*

Detailed methods for TMA construction have been described elsewhere [6]. Briefly, paraffin-embedded tumor blocks were requested from clinical pathology facilities. Study pathologists (JG, TK) marked hematoxylin & eosin (H&E)-stained slides to indicate areas enriched for invasive breast cancer for coring, and TMA construction and sectioning were carried out at the Translational Pathology Lab (TPL), University of North Carolina at Chapel Hill (UNC) for CBCS and at Roswell Park Cancer Institute (RPCI) for BWHS & WCHS. The resulting TMA blocks contained tumor cores measuring either 1.0 mm (CBCS) or 0.6 mm in diameter (BWHS and WCHS). Unstained slides were requested from clinical pathology facilities when tumor blocks were not available. Unstained slides and TMA sections were stored at TPL in a nitrogen desiccation chamber prior to IHC staining.

All immunohistochemistry (IHC) was performed at the UNC TPL under pathologist supervision (JG, TK) using the Bond fully-automated slide staining system (Leica Microsystems Inc., Norwell, MA). Detailed methods for IHC staining of ER, PR and HER2 have been described elsewhere [6]. For Ki67, EGFR and CK5/6, slides were deparaffinized in Bond Dewax solution (AR9222) and hydrated in Bond Wash solution (AR9590). Antigen retrieval was performed at 100°C for 30 min in Bond-epitope retrieval solution 1 at pH 6.0 (AR9961). Slides were incubated for 30 minutes at room temperature with primary antibody against EGFR (1:300; clone EP22; Epitomics (Burlingame, CA)) or for 15 minutes at room temperature with primary antibody against CK5/6 (1:50; clone D5/16B4; Dako (Carpinteria, CA)) or Ki67 (1:50; clone MIB1; Dako (Carpinteria, CA)). Antibody detection of EGFR and Ki67 was performed using the Bond Polymer Refine Detection System (DS9800) and the Bond Intense R Detection System (DS9263) supplemented with Dako EnVision Mouse (K4001) was used for detection of CK5/6. Negative controls (without primary antibody) were also included with each staining batch. Stained slides were digitally imaged at 20× magnification using the Aperio ScanScope XT (Aperio Technologies, Vista, CA) and digital images were stored in the Aperio Spectrum Database at TPL.
