# Supplementary material for: Frequency of breast cancer subtypes among African American women in the AMBER consortium
Source: Breast Cancer Res. 2018 Feb 6;20:12. doi: 10.1186/s13058-018-0939-5 (PMC5801839; doi:10.1186/s13058-018-0939-5)
Supplement: Supplementary file 2 — Receiver operating characteristic (ROC) curve in 250 HER2-negative luminal cases (A) and 309 luminal cases (B) regardless of HER2 status, using Ki67 expression to identify PAM50-based luminal B cases. A Ki67 threshold of 7.6% among HER2-negative luminal cases (A) and 7.1% among all luminal cases (B) maximized sensitivity and specificity for identifying PAM50-based luminal B tumors. AUC, area under the curve. (DOCX 43 kb) [file 13058_2018_939_MOESM2_ESM.docx]

**
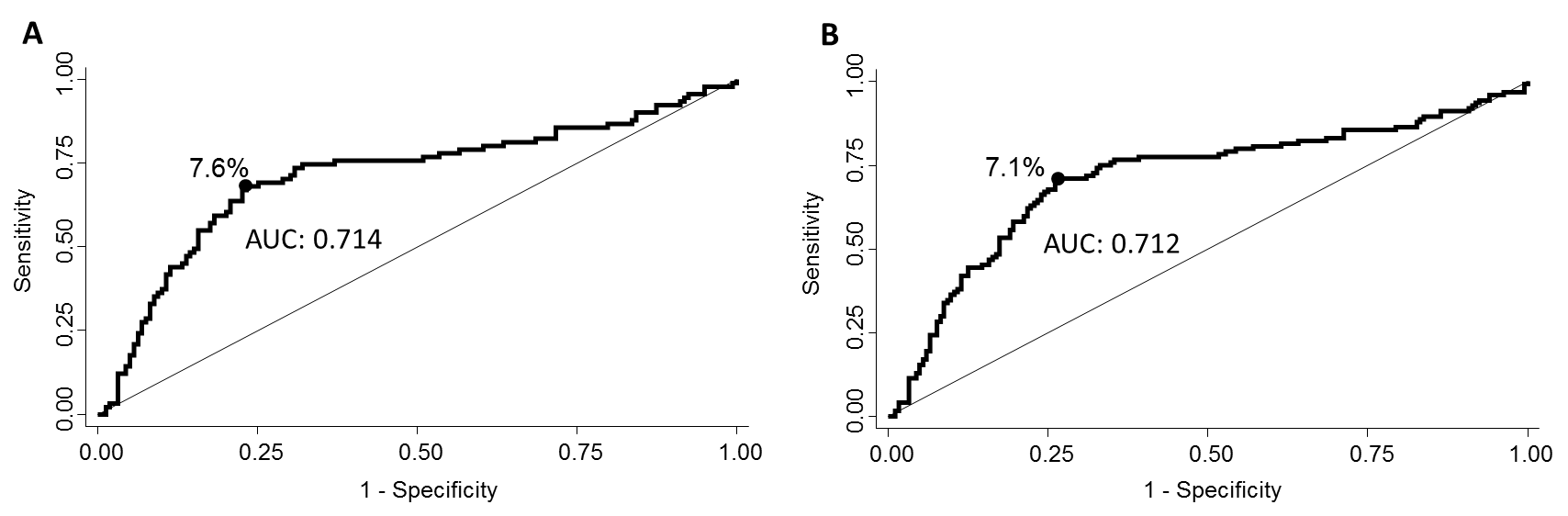
**

**Supplementary Figure 1:** Receiver operating characteristic (ROC) curve in A) 250 HER2-negative Luminal cases and B) 309 Luminal cases regardless of HER2 status, using Ki67 expression to identify PAM50-based luminal B cases. A Ki67 threshold of A) 7.6% among HER2-negative Luminal cases and B) 7.1% among all Luminal cases maximized sensitivity and specificity for identifying PAM50-based luminal B tumors. AUC=area under the curve
