# Supplementary material for: Frequency of breast cancer subtypes among African American women in the AMBER consortium
Source: Breast Cancer Res. 2018 Feb 6;20:12. doi: 10.1186/s13058-018-0939-5 (PMC5801839; doi:10.1186/s13058-018-0939-5)
Supplement: Supplementary file 3 — Classification of luminal breast cancer cases using data from medical records in the AMBER consortium. (DOCX 12 kb) [file 13058_2018_939_MOESM3_ESM.docx]

**Supplementary Table 1:** Classification of Luminal breast cancer cases using data from medical records in the AMBER consortium

|  | | **Subtype frequency, n (%)** | **Sensitivity for PAM50 subtype** | **Specificity for PAM50 subtype** | **Accuracy for PAM50 subtype** |
| --- | --- | --- | --- | --- | --- |
| **HR/HER2** | |  |  |  |  |
| Luminal A | HR+/HER2- | 670 (55) | 81% | 68% | 72% |
| Luminal B | HR+/HER2+ | 143 (12) | 28% | 92% | 79% |
| **HR/HER2/combined grade** | |  |  |  |  |
| Luminal A | HR+/HER2-, low/intermediate grade | 484 (40) | 67% | 86% | 80% |
| Luminal B | HR+/HER2+, *or* HR+/HER2-, high grade | 329 (27) | 66% | 81% | 78% |
| **HR/combined grade** | |  |  |  |  |
| Luminal A | HR+, low/intermediate grade | 537 (44) | 74% | 83% | 80% |
| Luminal B | HR+, high grade | 209 (17) | 57% | 88% | 81% |

HR=hormone receptor
