# Supplementary material for: Frequency of breast cancer subtypes among African American women in the AMBER consortium
Source: Breast Cancer Res. 2018 Feb 6;20:12. doi: 10.1186/s13058-018-0939-5 (PMC5801839; doi:10.1186/s13058-018-0939-5)
Supplement: Supplementary file 4 — Kernel density plots showing the distribution of Ki67 and PR expression (A and B, respectively) in PAM50-defined luminal A (n = 159) and luminal B (n = 91) subtypes. Analyses were restricted to IHC-based HER2-negative tumors. Vertical lines indicate an 8% threshold for Ki67 (A) and a 20% threshold for PR (B). (DOCX 56 kb) [file 13058_2018_939_MOESM4_ESM.docx]

**
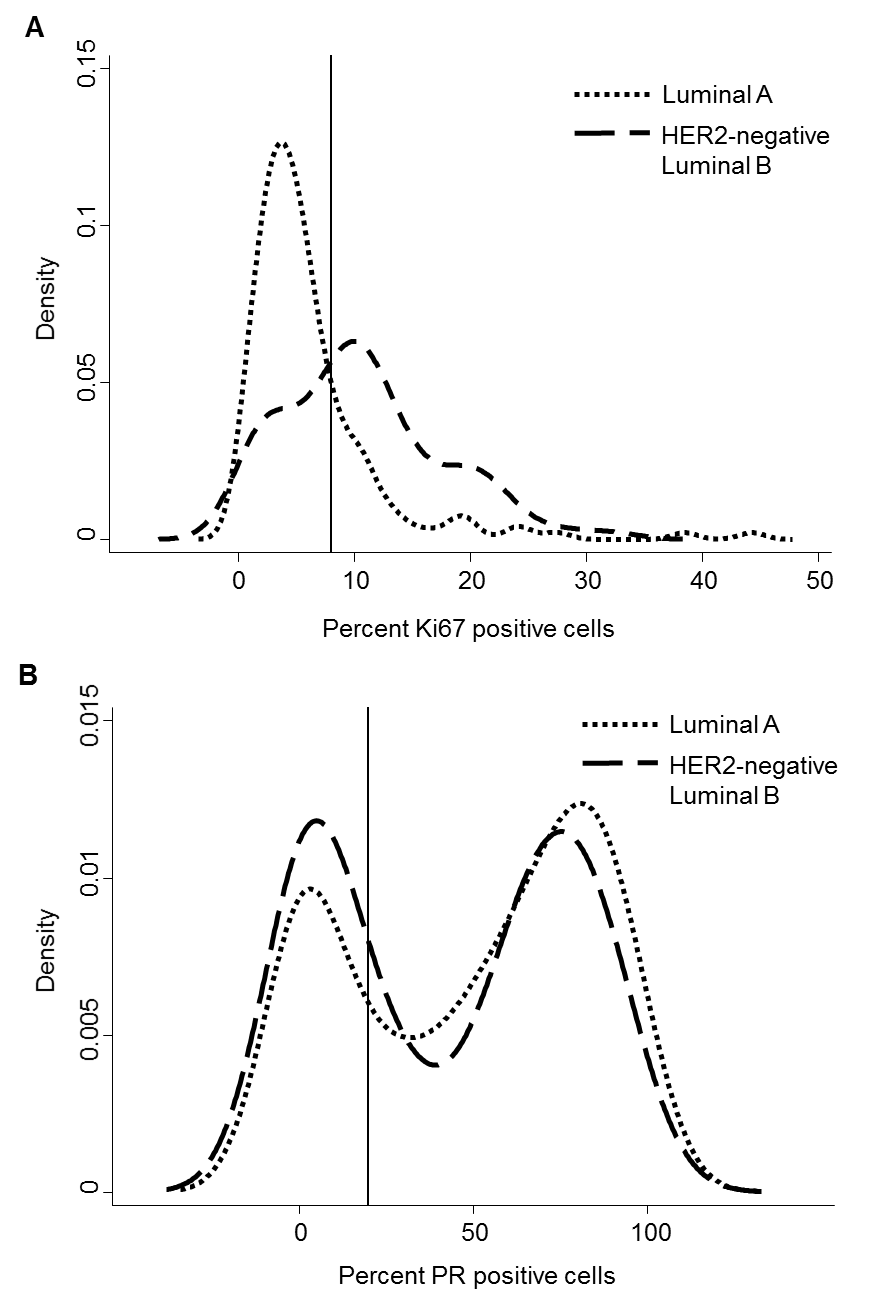
**

**Supplementary Figure 2:** Kernel density plots showing the distribution of Ki67 and PR expression (A and B, respectively) in PAM50-defined luminal A (n=159) and luminal B (n=91) subtypes. Analyses were restricted to IHC-based HER2-negative tumors. Vertical lines indicate an 8% threshold for Ki67 (A) and a 20% threshold for PR (B).
