# Supplementary material for: Frequency of breast cancer subtypes among African American women in the AMBER consortium
Source: Breast Cancer Res. 2018 Feb 6;20:12. doi: 10.1186/s13058-018-0939-5 (PMC5801839; doi:10.1186/s13058-018-0939-5)
Supplement: Supplementary file 5 — Effect of raising the Ki67 threshold on the performance of the IHC-based luminal classification scheme. (DOCX 12 kb) [file 13058_2018_939_MOESM5_ESM.docx]

**Supplementary Table 2:** Effect of raising the Ki67 threshold on the performance of the IHC-based Luminal classification scheme

| **IHC-based classification scheme** | | **Subtype frequency, n (%)** | **Sensitivity** | **Specificity** | **Accuracy** |
| --- | --- | --- | --- | --- | --- |
| Luminal A | HR+, <10% Ki67 | 689 (48) | 78% | 77% | 78% |
| Luminal B | HR+, ≥10% Ki67 | 218 (15) | 47% | 91% | 82% |
| Luminal A | HR+, <14% Ki67 | 797 (55) | 86% | 70% | 75% |
| Luminal B | HR+, ≥14% Ki67 | 110 (8) | 25% | 95% | 80% |

HR=hormone receptor
