# Supplementary material for: Frequency of breast cancer subtypes among African American women in the AMBER consortium
Source: Breast Cancer Res. 2018 Feb 6;20:12. doi: 10.1186/s13058-018-0939-5 (PMC5801839; doi:10.1186/s13058-018-0939-5)
Supplement: Supplementary file 6 — Odds ratios and 95% confidence intervals for age and menopauseb status at diagnosis by six-marker immunohistochemistry-defined subtypea in the AMBER consortium. (DOCX 13 kb) [file 13058_2018_939_MOESM6_ESM.docx]

**Supplementary Table 3:** Odds ratios and 95% confidence intervals for age and menopause^b^ status at diagnosis by six-marker immunohistochemistry-defined subtype^a^ in the AMBER consortium

|  | **≥50 yrs,**  **N (%)** | **<50 yrs,**  **N (%)** | **OR^b^ (95% CI)** | **Postmeno,**  **N (%)** | **Premeno,**  **N (%)** | **OR^b^ (95% CI)** |
| --- | --- | --- | --- | --- | --- | --- |
| Luminal A | 305 (39) | 207 (34) | 1 | 300 (39) | 204 (35) | 1 |
| Luminal B | 195 (25) | 138 (23) | 0.97 (0.73-1.29) | 194 (25) | 135 (23) | 1.01 (0.76-1.35) |
| ER-/HER2+ | 49 (6) | 62 (10) | 1.86 (1.23-2.83) | 51 (7) | 57 (10) | 1.64 (1.08-2.49) |
| Basal-like | 224 (29) | 201 (33) | 1.26 (0.97-1.64) | 233 (30) | 182 (31) | 1.14 (0.87-1.48) |
| *p-value* | *0.008* | |  | *0.107* | |  |
| Unclassified | 93 | 78 |  | 93 | 72 |  |

^a^Luminal A: HR+, low Ki67; Luminal B: HR+, high Ki67; ER-/HER2+: ER <10% and HER2-positive; Basal-like: HR-, HER2- and (EGFR+ *or* CK5/6+).

Tumors with five-marker negative (n=7), HER2-equivocal (n=81), and missing biomarker (n=83) status remained unclassified.

^b^n=25 cases were missing menopause status

^c^ORs are adjusted for AMBER study

chi-square test p value excludes unclassified cases
